# Quantification of Sodium Relaxation Times and Concentrations as Surrogates of Proteoglycan Content of Patellar CARTILAGE at 3T MRI

**DOI:** 10.3390/diagnostics11122301

**Published:** 2021-12-08

**Authors:** Benedikt Kamp, Miriam Frenken, Jan M. Henke, Daniel B. Abrar, Armin M. Nagel, Lena V. Gast, Georg Oeltzschner, Lena M. Wilms, Sven Nebelung, Gerald Antoch, Hans-Jörg Wittsack, Anja Müller-Lutz

**Affiliations:** 1 Department of Diagnostic and Interventional Radiology, Medical Faculty, University Dusseldorf, D-40225 Dusseldorf, Germany; Benedikt.Kamp@med.uni-duesseldorf.de (B.K.); JanMartin.Henke@med.uni-duesseldorf.de (J.M.H.); Daniel.Abrar@med.uni-duesseldorf.de (D.B.A.); Lena.Wilms@med.uni-duesseldorf.de (L.M.W.); Sven.Nebelung@med.uni-duesseldorf.de (S.N.); Antoch@med.uni-duesseldorf.de (G.A.); Hans-Joerg.Wittsack@med.uni-duesseldorf.de (H.-J.W.); Anja.Lutz@med.uni-duesseldorf.de (A.M.-L.); 2 Clinic of Nuclear Medicine, Medical Faculty, University Dusseldorf, D-40225 Dusseldorf, Germany; 3 Institute of Radiology, University Hospital Erlangen, Friedrich-Alexander-Universität Erlangen-Nürnberg (FAU), D-91054 Erlangen, Germany; Armin.Nagel@uk-erlangen.de (A.M.N.); Lena.Gast@extern.uk-erlangen.de (L.V.G.); 4 German Cancer Research Center (DKFZ), Division of Medical Physics in Radiology, D-69120 Heidelberg, Germany; 5 Russell H. Morgan Department for Radiology and Radiological Science, The Johns Hopkins University School of Medicine, Baltimore, MD 21205-2196, USA; goeltzs1@jhmi.edu; 6 F. M. Kirby Research Center for Functional Brain Imaging, Kennedy Krieger Institute, Baltimore, MD 21205-2196, USA

**Keywords:** Sodium MRI, ^23^Na, sodium relaxation times, knee, cartilage, fluid suppression, inversion recovery, biochemical imaging, proteoglycan

## Abstract

Sodium MRI has the potential to depict cartilage health accurately, but synovial fluid can influence the estimation of sodium parameters of cartilage. Therefore, this study aimed to reduce the impact of synovial fluid to render the quantitative compositional analyses of cartilage tissue technically more robust. Two dedicated protocols were applied for determining sodium $T_{1}$ and $T_{2}^{*}$ relaxation times. For each protocol, data were acquired from 10 healthy volunteers and one patient with patellar cartilage damage. Data recorded with multiple repetition times for $T_{1}$ measurement and multi-echo data acquired with an additional inversion recovery pulse for $T_{2}^{*}$ measurement were analysed using biexponential models to differentiate longitudinal relaxation components of cartilage ($T_{1,\mathrm{car}}$) and synovial fluid ($T_{1,\mathrm{syn}}$), and short ($T_{2s}^{*}$) from long ($T_{2l}^{*}$) transversal relaxation components. Sodium relaxation times and concentration estimates in patellar cartilage were successfully determined: $T_{1,\mathrm{car}}$ = 14.5 ± 0.7 ms; $T_{1,\mathrm{syn}}$ = 37.9 ± 2.9 ms; c($T_{1}$-protocol) = 200 ± 48 mmol/L; $T_{2s}^{*}$ = 0.4 ± 0.1 ms; $T_{2l}^{*}$ = 12.6 ± 0.7 ms; c($T_{2}^{*}$-protocol) = 215 ± 44 mmol/L for healthy volunteers. In conclusion, a robust determination of sodium relaxation times is possible at a clinical field strength of 3T to quantify sodium concentrations, which might be a valuable tool to determine cartilage health.

## 1. Introduction

Joint cartilage serves as a mechanical buffer and plays a pivotal role in the joint’s health and functionality. Therefore, cartilage loss is a hallmark change in the pathogenesis of several degenerative and inflammatory joint diseases such as osteoarthritis, rheumatoid arthritis and psoriatic arthritis and is associated with pronounced disease burden and functional disability [1,2,3,4]. While some easier to implement techniques like ^1^H T_2_ mapping have been shown to be a feasible tool for differentiating healthy and degraded cartilage, they detect mainly structural changes in the collagen matrix of cartilage [5]. However, prior to the manifestation of irreversible structural cartilage degradation, early and potentially reversible changes in cartilage composition occur and proteoglycans (PG) are lost. For the detection of compositional changes, techniques with high PG specificity such as delayed gadolinium-enhanced MRI, glycosaminoglycan (GAG) chemical exchange saturation transfer or sodium (^23^Na) MRI have emerged [4,6,7,8,9,10,11,12,13].

The GAG side chains of PGs consist of carboxyl and sulphate groups and create a fixed charge density (FCD) [14]. The negatively charged FCD attracts positively charged ^23^Na ions, which can be measured by ^23^Na MRI and, consequently, the intra-tissue ^23^Na concentration indicates the PG content [15]. Therefore, the ^23^Na signal can be used to assess the compositional make-up of cartilage and to detect PG depletion as a sign of early degeneration. Beyond ^23^Na concentrations, ^23^Na relaxation times of cartilage change with degeneration, as shown in enzymatically degraded cartilage samples [16].

Despite its high sensitivity for PGs, the signal-to-noise ratio (SNR) of ^23^Na MRI of cartilage is up to three orders of magnitude lower compared to conventional proton MRI [17]. This is due to lower abundance of ^23^Na as compared to hydrogen in humans and the generally lower nuclear magnetic resonance sensitivity [18]. Transverse relaxation of the ^23^Na signal in cartilage follows a biexponential decay with a short ($T_{2s}^{*}$) and a long component ($T_{2l}^{*}$). The already low SNR and fast decay of the ^23^Na signal with increasing echo times (TEs) requires the use of ultra-short echo-time (UTE) sequences [19]. ^23^Na MRI is usually performed with lower spatial resolution than conventional proton MRI to keep image acquisition periods manageable, yet this comes at the expense of more and more severe partial volume effects (PVEs). In articular cartilage, the ^23^Na signal from the synovial fluid that surrounds the cartilage spills into the cartilage signal, thereby leading to inaccurate quantification of ^23^Na concentration and relaxation times [20]. For ^23^Na MRI on clinical 3T MRI scanners (that incurs lower SNR and more PVEs as compared to ≥7T MRI scanners [21]), this aspect becomes very relevant as it challenges correct classification of pixels as cartilage or synovial fluid and artificially increases $T_{1}$ and $T_{2}^{*}$ relaxation times of patellar cartilage. Therefore, methods to reduce the influence of synovial fluid on ^23^Na relaxation times and concentrations, especially in regions prone to PVEs, are highly desirable to advance the clinical applicability of ^23^Na MRI in cartilage assessment.

Human patellar cartilage ^23^Na $T_{1}$ ($T_{1,\mathrm{car}}$ = 21 ms) and $T_{2}^{*}$ relaxation times ($T_{2s}^{*}$ = 0.8 ms; $T_{2l}^{*}$ = 19.7 ms; biexponential model) have been shown to be shorter than those of synovial fluid ($T_{1,\mathrm{syn}}$ = 48 ms; $T_{2}^{*}$ = 47 ms, monoexponential model) when imaged at 4.7T and a high in-plane resolution of 1.5 × 1.5 mm^2^ [20]. However, even at this superior resolution, clear differentiation of cartilage and synovial fluid was only possible in certain areas adjacent to the joint capsule and not possible for the patellar cartilage. A potential way to suppress the signal of synovial fluid is the application of an inversion pulse to null the synovial fluid signal, which was introduced previously and can easily be applied for $T_{2}^{*}$ measurements [22,23,24]. However, the determination of $T_{1}$ with additional inversion pulses is challenging, as these directly interfere with cartilage $T_{1}$ relaxation.

In this study, we set out (1) to determine ^23^Na $T_{1}$ and $T_{2}^{*}$ relaxation times of patellar cartilage in the presence of PVEs at 3T. We aimed to develop a robust method for $T_{1}$ measurements of cartilage and to evaluate $T_{2}^{*}$ while using an inversion recovery (IR) method for fluid suppression. We then (2) aimed to apply the measured relaxation times to determine ^23^Na concentration maps. We hypothesized, in addition to successfully estimating the above ^23^Na parameters, to be able to observe a trend towards different relaxation times and concentrations between healthy controls and patients.

## 2. Materials and Methods

This study was conducted with two separate MRI protocols for measuring ^23^Na $T_{1}$ (protocol 1) and $T_{2}^{*}$ (protocol 2). Although it would be preferable to measure both $T_{1}$ and $T_{2}^{*}$ in a single MRI session, the approach with separate protocols ensures that more data points can be acquired and thus, more stable results should be obtained for the estimation of $T_{1}$ and $T_{2}^{*}$ parameters.

### 2.1. Study Population

Two age-matched cohorts of healthy volunteers were examined, one for each study protocol ($T_{1}$ protocol: 4 females, 6 males, mean age 23 ± 3 years, minimum/maximum 19/29 years; $T_{2}^{*}$ protocol: 3 females, 7 males, mean age 23 ± 2 years, minimum/maximum 19/28 years). Participants were excluded from the healthy volunteer group if any degenerative joint disease of the knee or cartilage damage was known. Participants were also excluded if they reported a history of acute or chronic knee pain as well as previous surgery to the index knee.

**Figure 1:**
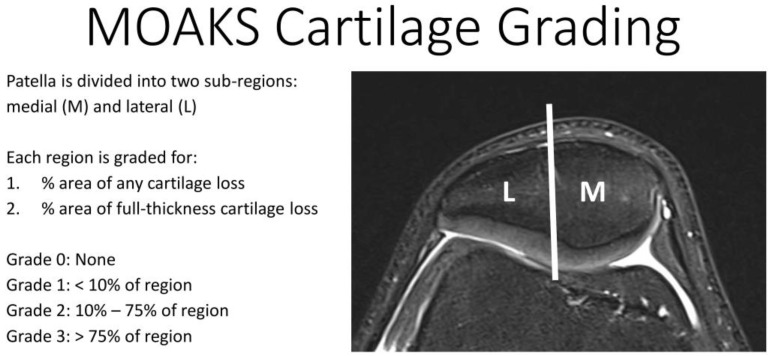
MRI Osteoarthritis Knee Score (MOAKS). This system divides cartilage surfaces within the retropatellar knee into the lateral (L) and the medial (M) subregions. Cartilage lesions in each subregion are then analyzed using scores based on the amount of any cartilage loss as a percentage of the subregion and amount of full-thickness cartilage loss as percentage of subregion.

Two patients with retropatellar chondropathy, one for each protocol, were studied using the same protocols as above. For the $T_{1}$ protocol, one 66 years old female patient with established osteochondrosis of the left knee was included. Following the MRI Osteoarthritis Knee Score (MOAKS) [25], which classifies cartilage lesions from 0 (no defects) to 3 (severe defects) according to the percentage extent of any defect and the percentage extent of full-thickness cartilage defects (Figure 1), their patellar cartilage lesions were classified as grade 2/1 (any/full-thickness) in the medial retropatellar region and 2/0 (any/full-thickness) in the lateral subregion. For the $T_{2}^{*}$ protocol, a 30-year-old female patient was included with posttraumatic cartilage defects of the right knee, which were classified as grade 3/1 (any/full-thickness) in the medial and grade 1/1 (any/full-thickness) in the lateral subregion according to the MOAKS classification.

The study was approved by the local ethics committee (Ethics Committee, Medical Faculty of the Heinrich-Heine-University Düsseldorf, study number 4733R), and written informed consent was obtained from all volunteers and patients.

### 2.2. MRI

All images were acquired using a 3T MRI scanner (Siemens MAGNETOM Prisma, Siemens Healthineers, Erlangen, Germany) and a dual-tuned ^23^Na/^1^H surface coil (RAPID Biomedical GmbH, Rimpar, Germany) with a 11 cm circular ^23^Na resonator and a 18 cm × 24 cm rectangular ^1^H resonator. Once all participants were placed in the feet-first and supine position, the dual-tuned coil was placed on top of the right knee of healthy volunteers and on top of the affected knee of patients. For reference purposes, patients were also studied using a dedicated ^1^H 15-channel knee coil (Tx/Rx Knee 15 Flare Coil, Siemens Healthineers, Erlangen, Germany) in line with clinical standard routines. Consequently, the coil had to be replaced between the ^23^Na and ^1^H measurements.

For quantification of ^23^Na concentrations, three cylindrical phantoms (diameter: 1.5 cm, length: 10 cm) with different ^23^Na concentrations (50 mmol/L, 100 mmol/L and 200 mmol/L) and a fixed agarose content of 4% (ROTI^®^Garose, Carl ROTH GmbH & Co. KG, Karlsruhe, Germany) were manufactured by B.K. and attached medial to the examined knee [11,23,26]. All ^23^Na MRI was performed using a density-adapted 3D radial sequence (DA-3D-RAD), which was developed by Nagel et al. and has been shown to improve SNR compared to conventional 3D radial sequences through more efficient k-space sampling [27]. Furthermore, inversion recovery pulses and spoiler gradients are usable with this sequence.

#### 2.2.1. Characterization of the ^23^Na Coil

The sensitivity of the ^23^Na coil was evaluated by measuring a homogenous water phantom and the sensitivity image was then used to correct the ^23^Na images of all participants on a pixel-by-pixel basis [28]. The phantom was of cylindrical shape (diameter: 18 cm; height: 7.5 cm) and filled with 100 mmol/L NaCl solution.

To assess B_1_ homogeneity of the surface coil for ^23^Na MRI, the field dependency was mapped with the same 100 mmol/L NaCl phantom as above using the double-angle method [29,30]. To this end, two ^23^Na images with varying FAs were acquired. The applied sequence parameters for these measurements are summarized in Table 1.

#### 2.2.2. MRI Sequence Parameters

Two separate study protocols were designed for relaxation time measurements. For both protocols, a ^1^H localizer sequence and two sequences for the adjustment of the required reference voltage were acquired first, one for ^1^H MRI and one for ^23^Na MRI [31]. Manual B_0_ shimming was performed prior to DA-3D-RAD image acquisition. High-resolution ^1^H MRI was performed using the DA-3D-RAD sequence as the anatomic reference. The parameters for ^23^Na MRI with the DA-3D-RAD differed between protocol 1 and protocol 2 as summarized in Table 1.

For ^23^Na $T_{1}$ determination images were acquired with 17 different TR to obtain a high number of data points for the later explained fitting procedure. For ^23^Na $T_{2}^{*}$ determination a multi-echo DA-3D-RAD was acquired three times with four different TEs each time, resulting in 12 data points. This interleaving acquisition pattern of different TEs with multiple sequences was chosen because the readout time would not allow shorter spacing between TE per single sequence. To minimize synovial fluid-induced PVEs and their bearing on $T_{2}^{*}$ estimation, a rectangular inversion recovery pulse with an inversion time of TI = 24 ms and an inversion pulse duration of 1 ms was used to null the synovial fluid signal [11,24,32].

For clinical reference purposes, patients were further examined using a conventional knee coil with proton density (PD)-weighted fat-saturated and $T_{1}$-weighted sequences. The parameters for the additional sequences to study the patients are detailed in Table 2.

### 2.3. Image Post-Processing

The images were reconstructed using a Hann Filter to increase SNR and reduce Gibbs ringing. The ^23^Na images were motion-corrected using the in-house developed software stroketool [33], which utilises a cross-correlation algorithm based on advanced normalization tools [34]. For further data evaluation and ROI definition, in-house developed MATLAB (MathWorks, Natick, MA, USA, R2018a) scripts were used. To correct displacement between the ^1^H and ^23^Na DA-3D-RAD images, regions-of-interest (ROIs) were drawn around the agarose phantoms in the ^1^H and one ^23^Na DA-3D-RAD image. The calculated shift between these ROIs was then used to overlay the ^1^H DA-3D-RAD image precisely with the ^23^Na image.

Relaxation curve fitting was performed using the mean values of the cartilage ROI for more stable results than would be obtained with pixelwise fitting. The data of protocol 1 was fitted with a biexponential two-pool model as indicated in Equation (1) to determine $T_{1}$ for both cartilage ($T_{1,\mathrm{car}}$) and synovial fluid ($T_{1,\mathrm{syn}}$) from the signal intensity S(t):

(1) $$S\left(t\right)=S\left(0\right)\cdot \left(p_{\mathrm{car}}\cdot \left(1-e^{\frac{-t}{T_{1,\mathrm{car}}}}\right)+\left(1-p_{\mathrm{car}}\right)\cdot \left(1-e^{\frac{-t}{T_{1,\mathrm{syn}}}}\right)\right)+\mathrm{noise}$$

In this approach, $p_{\mathrm{car}}$ designates the fraction of the cartilage $T_{1,\mathrm{car}}$ of the total $T_{1}$ signal relaxation, while $\left(1-p_{\mathrm{car}}\right)$ is the fraction of synovial fluid, assuming that signal only comes from these two pools. The values of $p_{\mathrm{car}}$ follow the condition $0<p_{\mathrm{car}}<1$.

Data from protocol 2 were used to determine transverse relaxation times for the patellar cartilage using a validated biexponential model introduced by our group previously (Equation (2)), assuming a short and a long component $T_{2s}^{*}$ and $T_{2l}^{*}$ [31]:

(2) $$S\left(t\right)=S\left(0\right)\cdot \left(p_{s}\cdot e^{\frac{-t}{T_{2s}^{*}}}+\left(1-p_{s}\right)\cdot e^{\frac{-t}{T_{2l}^{*}}}\right)+\mathrm{noise}$$

Here, $p_{s}$ defines the fraction of the $T_{2s}^{*}$ signal decrease in the $T_{2}^{*}$ signal decrease as a whole, satisfying the condition $0<p_{s}<1$.

Finally, the sodium images were used to determine the sodium concentration in the retropatellar cartilage. Agarose phantoms for the quantification of sodium concentrations were manufactured as described previously [31]. The relaxation times of the phantoms were: $T_{1}$ = 38.6 ms; $T_{2s}^{*}$ = 4.5 ms; $T_{2l}^{*}$ = 15.3 ms; $p_{s}$ = 66.8%. The relaxation times were used to correct the influence of the relaxation time difference between agarose phantoms and cartilage on their signal ratio. For the in-vivo cartilage measurements acquired in this study, the arithmetic means of the determined longitudinal relaxation times for the group of volunteers measured with protocol 1 and the transverse relaxation times determined for the group of volunteers measured with protocol 2 were applied for this correction.

The corrected signal from the agarose phantoms was fitted linearly to calculate the ^23^Na concentration of the cartilage. Agarose phantoms were excluded if positioned too far from the surface coil causing insufficient excitation flip angles, which had been determined previously by B_1_ mapping in the course of the characterization of the ^23^Na coil. To estimate ^23^Na concentrations in cartilage, the partial volume correction method of previous studies of our group [31] was applied. The spatial dimensions of the patellar cartilage are comparable to and sometimes smaller than the voxel size of the ^23^Na images. When the voxel of the ^23^Na image is not completely filled with cartilage, the ^23^Na concentration of the cartilage in this voxel is often averaged with background noise, resulting in the underestimation of ^23^Na concentration in that voxel depending on how much volume of the voxel is filled with cartilage. To reduce this effect, the higher resolution of the ^1^H images and the ROIs drawn on those images are used to calculate the volume fraction of cartilage in each ^23^Na voxel. The volume fraction is averaged over the whole ROI and the concentration values in the ROI are multiplied by the inverse of this volume fraction. Finally, the resulting ^23^Na concentration estimates were divided by 0.75, because ~25% of cartilage is made up by solids that do not contribute to the ^23^Na signal [24,35,36].

^23^Na concentration maps were determined for both protocols. Specifically, for protocol 1 we selected the image acquired at a TR of 70 ms to minimize $T_{1}$ weighting of the ^23^Na signal. For protocol 2, the shortest available TE (TE = 0.3 ms) was selected to ensure high SNR and to minimize T_2_ weighting. The ROIs of the retropatellar cartilage were independently drawn by two experienced radiologists on the ^1^H DA-3D-RAD images. Radiologist #1 (M.F.; 5 years of experience in musculoskeletal imaging) defined the ROIs twice (i.e., two weeks apart to allow for sufficient washout) to allow for intra-reader reliability assessment, while radiologist #2 (D.B.A.; 5 years of experience in musculoskeletal imaging) defined them once for inter-reader reliability assessment.

Segmentations were performed using in-house developed MATLAB scripts by manually delineating the outer contours of the patellar cartilage in the ^1^H DA-3D-RAD images. Synovial fluid was not intentionally included in the ROIs; however, for the biexponential $T_{1}$ fitting described in Equation (1), it is expected to affect the signal in the ROIs anyway, because of PVEs caused by the large voxel size of the ^23^Na images and the close proximity of the synovial fluid to the cartilage.

### 2.4. Statistical Analysis

Statistical analysis was performed with SPSS (IBM Corp. Released 2020. IBM SPSS Statistics for Windows, Version 27.0. Armonk, NY: IBM Corp.). For the healthy controls, descriptive statistics (mean, standard deviation, minimum, median, maximum) were calculated for each protocol. For the relaxation times of the patients, the standard deviation was calculated across the respective measurements of the radiologists; the standard deviation of the ^23^Na concentration of the patients was determined across the pixelwise deviation of ^23^Na concentrations in the cartilage ROI.

To test whether the different settings (TE, TR, inversion pulse) in protocol 1 and 2 resulted in significant differences in ^23^Na concentration estimates, the mean concentration values between the volunteer groups were compared using the Wilcoxon signed-rank test with a significance level of *p* < 0.05. For assessing intra- and inter-reader reliability, single intraclass correlation coefficients (sICC) and average intraclass correlation coefficients (aICC) were calculated for the biexponential model parameters $T_{1,\mathrm{car}}$, $T_{1,\mathrm{syn}}$, $p_{\mathrm{car}}$, $T_{2s}^{*}$, $T_{2l}^{*}$, $p_{s}$ and ^23^Na concentrations of protocol 1 and 2 in healthy volunteers. The resulting intraclass correlation coefficients were categorized according to Koo et al. [37].

## 3. Results

**Figure 2:**
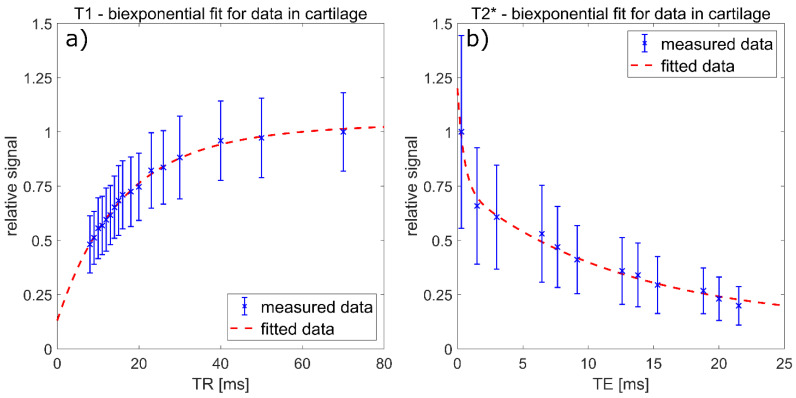
(**a**) Data points and biexponential fitting results to determine longitudinal ^23^Na relaxation times of the patellar cartilage of a representative healthy volunteer measured with protocol 1. The signal was normalized to the mean signal value at TR = 70 ms. The corresponding fitting results were: $T_{1,\mathrm{car}}$ = 14.1 ms; $T_{1,\mathrm{syn}}$ = 35.1 ms; $p_{\mathrm{car}}$ = 79.6%; $R^{2}$ = 0.9957. (**b**) Data points and biexponential fitting results to determine transverse ^23^Na relaxation times of the patellar cartilage of a representative healthy volunteer measured with protocol 2. The signal was normalized to the mean signal value at TE = 0.3 ms. The corresponding fitting results were $T_{2s}^{*}$ = 0.5 ms; $T_{2l}^{*}$ = 12.5 ms; $p_{s}$ = 41.0%; $R^{2}$ = 0.9906.

Relaxation time measurements were successfully performed on all participants. For the first measurement of radiologist #1, $T_{1}$ results were $T_{1,\mathrm{car}}$ = 14.45 ± 0.74 ms; $T_{1,\mathrm{syn}}$ = 37.91 ± 2.92 ms; $p_{\mathrm{car}}$ = 77.30 ± 3.73% for healthy volunteers measured with protocol 1 (Figure 2a) and $T_{1,\mathrm{car}}$ = 15.42 ± 0.08 ms; $T_{1,\mathrm{syn}}$ = 39.78 ± 0.14 ms; $p_{\mathrm{car}}$ = 71.23 ± 0.22% for the patient measured with protocol 1. Other associated $T_{1}$ results are summarized in Table 3. Intra- and inter-reader testing resulted in sICC($T_{1,\mathrm{car}}$) = 0.93; aICC($T_{1,\mathrm{car}}$) = 0.83; sICC($T_{1,\mathrm{syn}}$) = 0.94; aICC($T_{1,\mathrm{syn}}$) = 0.83; sICC($p_{\mathrm{car}}$) = 0.92 and aICC($p_{\mathrm{car}}$) = 0.69.

For $T_{2}^{*}$ results, the first measurements of radiologist #1 were $T_{2s}^{*}$ = 0.358 ± 0.147 ms; $T_{2l}^{*}$ = 12.62 ± 0.73 ms; $p_{s}$ = 34.39 ± 4.78% for healthy volunteers measured with protocol 2 (exemplary fit in Figure 2b) and $T_{2s}^{*}$ = 0.105 ± 0.001 ms; $T_{2l}^{*}$ = 13.99 ± 0.01 ms; $p_{s}$ = 25.86 ± 0.06% for the patient measured with protocol 2. Other associated $T_{2}^{*}$ results are summarized in Table 4. Intra- and inter-reader testing resulted in sICC($T_{2s}^{*}$) = 0.99; aICC($T_{2s}^{*}$) = 0.99; sICC($T_{2l}^{*}$) = 0.78; aICC($T_{2l}^{*}$) = 0.86; sICC($p_{s}$) = 0.99 and aICC($p_{s}$) = 0.98.

**Figure 3:**
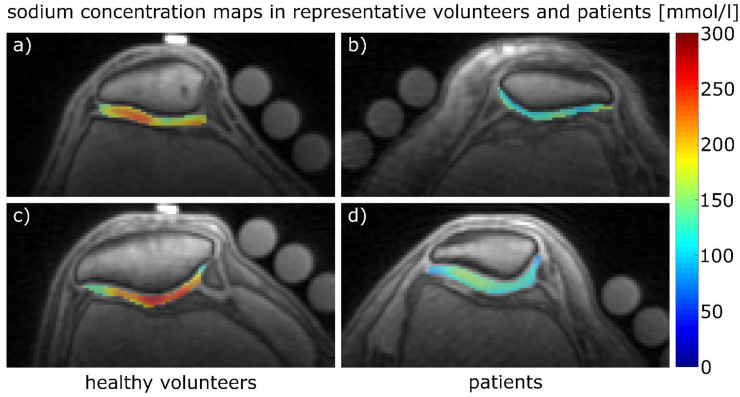
Color-coded ^23^Na concentration maps of the patellar cartilage (overlaid onto the corresponding ^1^H images). (**a**) Healthy volunteer (male, 22 years, right knee) measured with protocol 1. (**b**) Patient (female, 66 years, left knee) with moderate cartilage defects, some of which are severe on the medial side (MRI Osteoarthritis Knee Score (MOAKS): (2/1) medial, (2/0) lateral) measured with protocol 1. (**c**) Healthy volunteer (male, 25 years, right knee) measured with protocol 2. (**d**) Patient (female, 30 years, right knee) with extensive cartilage defects on the medial side and singular full-thickness cartilage defects on both sides (MOAKS: (3/1) medial, (1/1) lateral) measured with protocol 2. The corresponding mean ^23^Na concentration estimates were (**a**) 216 ± 42 mmol/L, (**b**) 158 ± 30 mmol/L, (**c**) 226 ± 64 mmol/L and (**d**) 135 ± 29 mmol/L. Partially visualized are agarose phantom tubes that were used for signal normalization. The scale on the right indicates ^23^Na concentration (mmol/L).

**Figure 4:**
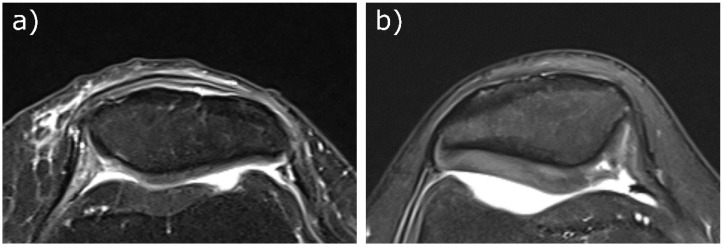
Transversal Proton Density-weighted fat-saturated images of the two patients. (**a**) Patient (female, 66 years, left knee, protocol 1) with moderate long-distance cartilage loss medial and lateral (10–75% of the subregion) and medial short-distance (<10% of the subregion) full-thickness defect (MRI Osteoarthritis Knee Score (MOAKS): (2/1) medial, (2/0) lateral). (**b**) Patient (female, 30 years, right knee, protocol 2) with very long-distance cartilage loss medial (>75% of the subregion) with short-stretch full-thickness defect (<10% of the subregion) and short-stretch, full-thickness lateral defect (<10% of the subregion), the latter is not visible in the depicted slice (MOAKS: (3/1) medial, (1/1) lateral).

The ^23^Na concentrations estimated from the first measurement of radiologist #1 for protocol 1 were 200 ± 48 mmol/L for the healthy volunteers (n = 10) and 158 ± 30 mmol/L for the patient, while for protocol 2, the values were 215 ± 44 mmol/L for the healthy volunteers (n = 9) and 135 ± 29 mmol/L for the patient. Proton images overlaid with ^23^Na concentration maps for volunteers and patients are shown in Figure 3. Transversal Proton Density-weighted fat-saturated images of the two patients are shown in Figure 4.

The ^23^Na concentration estimation results from all remaining participants are summarized in Table 5. Intra- and inter-reader testing resulted in sICC(protocol 1) = 0.94; aICC(protocol 1) = 0.78; sICC(protocol 2) = 0.90 and aICC(protocol 2) = 0.87. The concentration estimates between the healthy volunteer groups acquired for protocol 1 or 2 did not differ significantly (first measurement of radiologist #1: *p*-value= 0.441, data of remaining radiologist measurements in Table 5).

## 4. Discussion

In this study, we successfully determined (1) ^23^Na relaxation times of the patellar cartilage at a clinical field strength of 3T while minimizing the effects of PVEs caused by coarse spatial resolution and (2) calculated ^23^Na concentrations based on the relaxation times. The results of the sICC and aICC indicated good-to-excellent (0.78–0.99) intra- and moderate-to-excellent (0.69–0.99) inter-reader reliability for these parameters. We observed decreased $T_{2s}^{*}$ and increased $T_{2l}^{*}$ and $T_{1,\mathrm{car}}$ relaxation times in two patients with patellar cartilage degeneration as compared to healthy volunteers. However, further studies with more participants are necessary to draw more definitive conclusions.

In efforts to render such quantitative studies more reliable, the influence of synovial fluid on relaxation time quantification needs to be reduced. If not well controlled, the variable contribution of synovial fluid to the observed signal may confound ^23^Na quantification and further challenge the differentiation of healthy volunteers and patients with patellar cartilage degeneration or osteoarthritis. We successfully demonstrated the feasibility of using an inversion pulse for fluid suppression in $T_{2}^{*}$ determination and biexponential modeling for $T_{1}$ determination.

To our knowledge, applying a biexponential two-pool model to the ^23^Na signal for $T_{1}$ determination has not been performed before in this context. To our mind, it has distinct advantages. First, it reduces the influence of synovial fluid on relaxation time measurements in cartilage by including $T_{1,\mathrm{syn}}$ as fitting parameter in Equation (1), thus separating the longitudinal relaxation times of cartilage and synovial fluid. In widely used monoexponential one-pool models, these parameters would otherwise be merged in a singular parameter $T_{1}$, hampering accurate depiction of $T_{1}$ in cartilage. Second, it allows $T_{1,\mathrm{syn}}$ of synovial fluid and the signal fraction $p_{\mathrm{car}}$ to be estimated as additional parameters. It remains to be seen whether these parameters are of diagnostic value. Furthermore, applying inversion pulses for fluid suppression also reduces the signal from cartilage, which may prove problematic at lower field strengths than 3T.

Determining the ^23^Na relaxation times of cartilage is not only useful for a more accurate estimation of ^23^Na concentration by correcting the difference in signal ratio between cartilage and reference phantoms caused by different relaxation behaviour, but the relaxation times themselves can be an indicator for cartilage health [16]. In the aforementioned study, relaxation times of bovine patellar cartilage specimens were measured at 2T before and after enzymatic degradation by trypsin exposure, causing defined PG depletion [38]. Lower PG content was associated with decreases in $T_{2s}^{*}$ and increases in $T_{2l}^{*}$ and $T_{1,\mathrm{car}}$, which is in good agreement with our findings. Because data regarding the ^23^Na relaxation times in degraded cartilage is scarce and we only measured one patient per protocol, the following discussion is centred around the measurements performed on our healthy volunteers. An excerpt of study results from different authors regarding ^23^Na relaxation times in patellar cartilage is shown in Table 6.

Madelin et al. carried out extensive testing of knee cartilage relaxation times at 7T [39]. Their $T_{2}^{*}$ estimates are comparable to our results, although acquired at higher field strength. Theoretically, $p_{s}$ should be 60%, assuming a one compartment model, based on quadrupole interactions without magnetic field inhomogeneities, but under experimental conditions in the presence of field inhomogeneities and different tissue properties, $p_{s}$ has been shown to deviate from the theoretical value of 60% [40]. Madelin et al. found slightly higher $T_{1,\mathrm{car}}$ estimates than we did, which might be due to them using a MRI system with higher field strength (7T) compared to us (3T) and $T_{1}$ rising with increasing field strength [41]. Another reason could be the differences in their used TR values for $T_{1}$ measurement (30 ms to 250 ms). We focused on lower TR values to increase the number of data points available for the biexponential model, particularly for the shorter $T_{1,\mathrm{car}}$.

Feldman et al. measured ^23^Na relaxation times of the patellar cartilage and synovial fluid at 4.7T, reporting slightly longer ^23^Na longitudinal and transversal relaxation times compared to ours [20]. This group elaborated on the difficulty of separating voxels containing pure cartilage signal and pure fluid signal, which might have led to slightly overestimated $T_{1,\mathrm{car}}$ and $T_{2l}^{*}$ relaxation times as compared to our estimates. In addition, the previously discussed reasons for higher $T_{1,\mathrm{car}}$ values because of different MRI scanner field strengths and different TR intervals for fitting apply here as well. Staroswiecki et al. determined ^23^Na transversal relaxation times by monoexponential fitting [21]. However, due to the low resolution of ^23^Na images, these results are likely confounded by partial volume effects secondary to synovial fluid, a bias that we sought to address in this study.

Relaxation time measurements have also been performed with triple quantum filtered (TQF) ^23^Na MRI, which inherently suppresses synovial fluid [42]. Reported values at 3T were slightly lower ($T_{2s}^{*}$ = 0.84 ± 0.06 ms; $T_{2l}^{*}$ = 9.59 ± 0.35 ms) compared to our estimates. However, TQF signal acquisition has its own challenges in even lower SNR [18] and additional imaging artefacts caused by off-resonance effects [43]. Against this background, UTE sequences are therefore generally preferred for in-vivo ^23^Na MRI [44].

Our calculated ^23^Na concentrations in patients with degenerative patellar cartilage were lower compared to the mean and median values across all healthy volunteers. However, only two patients were studied by us. Chang et al. showed that ^23^Na concentration correlates with PG content and lower PG content is a sign of early-to-moderate degeneration [15,18]. The reduced ^23^Na concentrations of our patients may therefore indicate ongoing cartilage degeneration, which—as an imaging finding—is not surprising, as both patients already had morphologic cartilage defects. Future studies should therefore focus on patients with cartilage at risk (secondary to patient -or joint level- factors such as obesity, malalignment or previous injury) to further evaluate our method in such patients.

Madelin et al. reported ^23^Na concentrations for healthy volunteers and patients with (volunteers: 220–270 mmol/L; patients: 170–200 mmol/L) and without (volunteers: 180–210 mmol/L; patients: 170–190 mmol/L) the use of an inversion pulse for synovial fluid suppression [24]. Chang et al. reported similar findings at 7T comparing cartilage tissue after surgical cartilage resurfacing with native cartilage tissue of another joint area, both with (surgery: 108.9 ± 29.8 mmol/L; native: 249.8 ± 44.6 mmol/L) and without (surgery: 177.8 ± 54.1 mmol/L; native: 172.2 ± 30.3 mmol/L) applying an inversion pulse [15]. Our ^23^Na concentrations in healthy volunteers and patients are in good agreement with these data.

When interpreting our results, some limitations must be taken into consideration. One limitation is the long acquisition time, currently precluding $T_{1}$ and $T_{2}^{*}$ measurements within a single clinical MRI study. Total study time for $T_{1}$ and $T_{2}^{*}$ determination were 60 and 45 min, respectively. For future studies with increased patient numbers, the total measurement time for each protocol should be reduced to less than 30 min, which could be achievable by measuring less TE/TR data points for fitting combined with advanced techniques for image reconstruction [45,46,47,48]. However, these methods and their capability of producing stable results for cartilage data will need to be evaluated carefully.

While the patellar cartilage of patients was examined with additional sequences for clinical reference, the patellar cartilage of healthy volunteers was assessed by asking for history of pain or surgery in the examined knee. To further reduce the possibility of falsely including participants with cartilage damage in the healthy volunteer group, clinical reference MRI could also be performed for this group.

Furthermore, our healthy controls were not age-matched to the patients. In the intervertebral disks, a correlation between increasing participant age and reduced GAG chemical exchange saturation transfer effect has been shown [49]. A similar dependency might be possible for PG in articular cartilage and in larger clinical studies patients and healthy controls should preferably be age-matched to reduce the possibility of it confounding results. Additionally, future studies investigating the relation between age and PG content in cartilage could be conducted for clarification of this problem.

Other limitations include the low resolution of ^23^Na MRI in general, which limits its applicability for detecting smaller cartilage lesions and may be difficult to implement in smaller joints such as the ankle. The ^23^Na surface coil allowed evaluation of the patellofemoral joint only, while the femorotibial joint is not assessable using the present setup. The size of the ^23^Na resonator limits the sensible detection of ^23^Na signal in more distant areas like the femorotibial joint. It is important to note that inhomogenities of the B_1_ field were only considered regarding the placement of the Agarose phantoms by B_1_ field mapping. Spatial fluctuation of the B_1_ field will also reduce the efficiency of the fluid suppression of the inversion pulse applied in protocol 2, which could be mitigated by using adiabatic inversion pulses [23].

## 5. Conclusions

Synovial fluid can confound the measurement of ^23^Na relaxation times and concentrations in cartilage due to partial volume effects, especially at clinical field strength (≤3T). Therefore, in this study, two different methods to reduce the influence of synovial fluid, the usage of an inversion pulse to determine ^23^Na $T_{2}^{*}$ and ^23^Na concentrations and the appliance of a biexponential two-pool model for determining ^23^Na $T_{1}$, were successfully applied in healthy volunteers and two pilot patients with patellar cartilage damage. While the clinical value is necessarily reliant on a larger clinical database and is still unclear, this study introduces more parameters to quantitatively assess the tissue’s proteoglycan content as the key surrogate marker of early cartilage degeneration.

## Figures and Tables

**Table 1:** Sequence parameters for ^23^Na coil characterisation (coil sensitivity and B_1_ mapping) and for participant measurements (protocol 1, protocol 2 and ^1^H imaging).

	^23^Na Coil Sensitivity	B_1_ Mapping	Protocol 1 $\left(T_{1}\;\mathrm{Protocol}\right)$	Protocol 2 $\left(T_{2}^{*}\;\mathrm{Protocol}\right)$	^1^H Imaging
Sequence type	DA-3D-RAD	DA-3D-RAD	DA-3D-RAD	DA-3D-RAD	DA-3D-RAD
Nucleus	^23^Na	^23^Na	^23^Na	^23^Na	^1^H
Orientation	tra	tra	tra	tra	tra
Repetition time (ms)	60	300	8/9/10/11/12/13/14/15/16/18/20/23/26/30/40/50/70	84	30
Echo time (ms)	0.3	0.3	0.3	(0.30/6.45/12.60/18.80) (1.50/7.65/13.80/20.00) (3.00/9.15/15.30/21.50)	0.8
Inversion time (ms)	-	-	-	24	-
Inversion pulse Duration (ms)	-	-	-	1	-
Field of View (mm)	180 × 180 × 180	180 × 180 × 180	180 × 180 × 180	180 × 180 × 180	180 × 180 × 180
Projections	50,000	50,000	9000	9000	9000
Pixel size (mm/px)	3 × 3 × 3	3 × 3 × 3	3 × 3 × 3	3 × 3 × 3	1 × 1 × 1
Flip angle (°)	90	40/80	90	90	10
Pulse duration (ms)	0.5	0.5	0.5	0.5	0.2
Readout time (ms)	5	5	5	5	1
Averages	12	2	1	1	1
Total examination time (h:min:s)	10:00:00	16:40:00	00:57:45	00:37:48	00:04:30

Abbreviations: tra: transversal, DA-3D-RAD: density-adapted 3D radial sequence.

**Table 2:** Additional sequences and their acquisition parameters applied for patients.

	PD-Weighted fs	$T_{1}-\mathrm{Weighted}$
Sequence type	TSE	TSE
Turbo Factor	38	109
GRAPPA	2	2
Orientation	cor/tra/sag	sag
Repetition time (ms)	4980	864
Echo time (ms)	42	13
Field of View (mm)	160 × 160	160 × 160
Image matrix (px)	512 × 512	512 × 512
Pixel size (mm/px)	0.3 × 0.3	0.3 × 0.3
Flip angle (°)	180	180
Slices	35	35
Slice thickness (mm)	3	3
Total examination time (min:s)	09:57	03:10

Abbreviations: cor: coronal, tra: transversal, sag: sagittal, PD: proton density, fs: fat saturated, TSE: turbospin echo, GRAPPA: generalized autocalibrating partial parallel acquisition.

**Table 3:** Longitudinal ^23^Na relaxation times of the patellar cartilage of 10 healthy volunteers (4 females, 6 males, mean age 23 ± 3 years) and one patient (female, age 66 years) measured with protocol 1.

	Radiologist/			Volunteers			Patient
	Measurement	Mean	Std	Min	Median	Max	
	1/1	14.45	0.74	13.24	14.47	15.33	15.42
$T_{1,\mathrm{car}}$ (ms)	1/2	14.58	0.74	13.24	14.78	15.36	15.60
	2/1	14.61	0.68	13.26	14.80	15.29	15.60
	1/1	37.91	2.92	35.07	37.79	44.24	39.78
$T_{1,\mathrm{syn}}$ (ms)	1/2	38.32	2.89	35.02	39.24	44.50	39.65
	2/1	38.88	2.86	35.07	39.24	43.81	39.99
	1/1	77.30	3.73	72.80	78.53	82.64	71.23
$p_{\mathrm{car}}$ (%)	1/2	76.54	3.73	72.71	75.34	82.60	71.03
	2/1	75.10	4.15	68.10	73.58	82.55	70.69
	1/1	0.9917	0.0045	0.9808	0.9926	0.9959	0.9829
$R^{2}$	1/2	0.9918	0.0040	0.9831	0.9932	0.9962	0.9907
	2/1	0.9918	0.0045	0.9806	0.9930	0.9971	0.9903

Abbreviations: std: standard deviation, min: minimum, max: maximum, $p_{\mathrm{car}}$: fraction of $T_{1,\mathrm{car}}$ of the total $T_{1}$ relaxation, $R^{2}$: coefficient of determination for $T_{1}$ fit.

**Table 4:** Transverse ^23^Na relaxation times of the patellar cartilage in 10 healthy volunteers (3 females, 7 males, mean age 23 ± 2 years) and one patient (female, age 30 years) measured with protocol 2.

	Radiologist/			Volunteers			Patient
	Measurement	Mean	Std	Min	Median	Max	
	1/1	0.358	0.147	0.103	0.353	0.663	0.105
$T_{2s}^{*}$ (ms)	1/2	0.365	0.155	0.104	0.370	0.677	0.107
	2/1	0.365	0.174	0.105	0.357	0.751	0.107
	1/1	12.62	0.73	11.30	12.55	13.74	13.99
$T_{2l}^{*}$ (ms)	1/2	12.77	0.70	11.35	12.78	13.74	14.00
	2/1	12.79	0.72	11.49	12.71	13.98	14.00
	1/1	34.39	4.78	25.35	34.48	41.44	25.86
$p_{s}$ (%)	1/2	34.11	4.92	25.37	34.28	41.72	26.00
	2/1	33.81	5.09	25.28	34.66	41.79	25.99
	1/1	0.9856	0.0088	0.9643	0.9879	0.9931	0.9623
R^2^	1/2	0.9870	0.0097	0.9631	0.9910	0.9960	0.9657
	2/1	0.9861	0.0093	0.9640	0.9888	0.9949	0.9650

Abbreviations: std: standard deviation, min: minimum, max: maximum, $p_{s}$: fraction of $T_{2s}^{*}$ of the total $T_{2}^{*}$ relaxation, $R^{2}$: coefficient of determination for $T_{2}^{*}$ fit.

**Table 5:** ^23^Na concentration estimates of the patellar cartilage of 10 healthy volunteers (4 females, 6 males, mean age 23 ± 3 years) and one patient (female, age 66 years) measured with protocol 1 (TE = 0.3 ms and TR = 70 ms) and of nine healthy volunteers (3 females, 7 males, mean age 23 ± 2 years) and one patient (female, age 30 years) measured with protocol 2 (TE = 0.3 ms and TR = 84 ms). The listed *p*-values indicate the results of Wilcoxon signed-rank tests comparing the mean values of the healthy volunteers of protocol 1 and 2. Note that one healthy volunteer from protocol 2 was excluded from quantification of ^23^Na concentration because two agarose phantoms had to be excluded, leading to an insufficient number of data points for the fitting of the phantoms.

	Radiologist/			Volunteers				Patient
	Measurement	Mean	Std	Min	Median	Max	*p*-Value	
Protocol 1	1/1	215	44	166	203	291	0.441	135
^23^Na-Conc.	1/2	204	40	169	187	276	0.859	129
(mmol/L)	2/1	218	52	151	203	297	0.374	136
Protocol 2	1/1	200	48	130	199	267	-	158
^23^Na-Conc.	1/2	194	45	134	188	261	-	152
(mmol/L)	2/1	204	39	134	201	291	-	136

Abbreviations: std: standard deviation, min: minimum, max: maximum, conc.: concentration.

**Table 6:** Summary of results for ^23^Na relaxation times in patellar cartilage and synovial fluid from different authors.

	Magnetic Field Strength of MRI Scanner (T)	^23^Na Relaxation Time Results for Patellar Cartilage and Synovial Fluid				

		$T_{1,car}\;(\mathrm{ms})$	$T_{1,syn}\;(\mathrm{ms})$	$T_{2s}^{*}\;(\mathrm{ms})$	$T_{2l}^{*}\;(\mathrm{ms})$	$p_{s}\;(\%)$
Madelin et al. [39]	7.0	17.7 ± 2.6	-	0.5 ± 0.1	11.4 ± 1.8	39 ± 4
Feldman et al. [20]	4.7	21 ± 1	48 ± 3	0.8 ± 0.2	19.7 ± 0.5	65 ± 12
Staroswiecki et al. [21]	7.0	-	-	-	13.2 ± 1.5	-
	3.0	-	-	-	15.5 ± 1.3	-

Abbreviations: $p_{s}$–fraction of $T_{2s}^{*}$ of the total $T_{2}^{*}$ relaxation.

## Data Availability

Data can be provided by the authors upon reasonable request.
